# Is zoonotic *Plasmodium vivax* malaria an obstacle for disease elimination?

**DOI:** 10.1186/s12936-022-04349-6

**Published:** 2022-11-17

**Authors:** Bárbara Aparecida Chaves, Denise Anete Madureira de Alvarenga, Matheus de Oliveira Costa Pereira, Marcelo Gordo, Emanuelle L. Da Silva, Edson Rodrigues Costa, Aline Souza de Menezes Medeiros, Igor José Martins Pedrosa, Daniela Brito, Maurício Teixeira Lima, Maria Paula Mourão, Wuelton M. Monteiro, Nikos Vasilakis, Cristiana Ferreira Alves de Brito, Gisely C. Melo, Marcus V. G. Lacerda

**Affiliations:** 1 Instituto de Pesquisas Clínicas Carlos Borborema, Fundação de Medicina Tropical Doutor Heitor, Vieira Dourado, Manaus, AM 69040-000 Brazil; 2 Universidade do Estado do Amazonas, Av. Carvalho Leal, 1777, Cachoeirinha, Manaus, AM 69065- 001 Brazil; 3 Programa de Pós-Graduação em Ciências da Saúde, Universidade Federal do Amazonas, Rua Afonso Pena, 1053, Centro, Manaus, AM 69020-160 Brazil; 4 Grupo de pesquisa em Biologia molecular e imunologia da Malária, Instituto René Rachou (IRR), Fiocruz, Belo Horizonte, MG 30190-009 Brazil; 5 Laboratório de Biologia da Conservação, Instituto de Ciências Biológicas, Projeto Sauim-de-Coleira, Universidade Federal do Amazonas, Avenida General Rodrigo Octávio Jordão Ramos, 6200, Coroado, Manaus, AM 69080-900 Brazil; 6 Department of Pathology, Center for Biodefense and Emerging Infectious Diseases, Center for Tropical Diseases, Institute for Human Infection and Immunity, University of Texas Medical Branch, Galveston, TX 77555 USA; 7 Instituto Leônidas & Maria Deane (ILMD), Fiocruz, Manaus, AM 69057-070 Brazil

**Keywords:** *Plasmodium vivax*, Malaria, Zoonotic cycle, Malaria elimination, Non-human primates

## Abstract

**Background:**

The groundwork for malaria elimination does not currently consider the potential of *Plasmodium* zoonotic cycles that involve non-human primates (NHPs) in sylvatic environments. Since vivax malaria is less responsive to control measures, finding *Plasmodium vivax* infected NHPs adds even more concern.

**Methods:**

Both Free-living monkeys in forest fragments inside the urban area and captive monkeys from a local zoo had blood samples tested for *Plasmodium* species.

**Results:**

In this study, among the Neotropical monkeys tested, three (4.4%), one captive and two free-living, were found to be naturally infected by *P. vivax.*

**Conclusion:**

This important finding indicates that it is necessary to estimate the extent to which *P. vivax* NHP infection contributes to the maintenance of malaria transmission to humans. Therefore, the discussion on wildlife conservation and management must be incorporated into the malaria elimination agenda.

## Background

The world is joining forces, though not just to control malaria cases, but also to try to eliminate this disease, which is an important cause of death in economically disadvantaged countries. The World Malaria Report 2020 [1] and the Global Technical Strategy for Malaria 2016–2030 [2] both list some biological challenges and have identified the threats involved in eliminating malaria, such as parasite resistance to anti-malarials, vector control and asymptomatic or undiagnosed people, and these are the focus of discussions. However, these discussions may be missing one important target, i.e., that of the wild animal reservoir.

*Plasmodium falciparum*, *Plasmodium malariae*, *Plasmodium ovale*, and *Plasmodium vivax* are the main parasites transmitted by mosquito vectors of the genus *Anopheles* and cause malaria in humans. This transmission cycle of malaria theoretically makes it easier to fight this disease since it does not involve a wild animal reservoir, compared to other pathogens that have a complex cycle because they can infect more than one vertebrate host. The evolution of malaria parasites highlights their propensity to switch hosts; both *P. vivax* and *P. falciparum* first arose as human pathogens after a host switch from great apes in Africa [3, 4]. It is common knowledge that the transmission of *P. vivax* and *P. falciparum* occurs between infected mosquitoes and humans; however, some research groups have identified the possibility of a malaria reservoir in non-human primates [5, 6]. Zoonotic malaria transmission occurs in forests environments, during the close contact between humans and the mosquitoes that feed on *Plasmodium*-infected non-human primates (NHPs), and this is increasing due to habitat destruction and human encroachment into NHP habitats [7].

*Plasmodium cynomolgi*, and mainly *Plasmodium knowlesi*, are parasites that typically infect forest monkeys in Asia, but they can also cause zoonotic malaria when humans enter this parasite cycle. Another two *Plasmodium* species that can cause zoonotic malaria in Latin America are *Plasmodium brasilianum* and *Plasmodium simium* [8–10]. These zoonotic infections can be misdiagnosed during microscopy because the morphology of these parasites is similar to that of *P. malariae* and *P. vivax*, respectively [3, 11]. These pairs of parasites, *P. brasilianum/P. malariae* and *P. simium/P.vivax*, also have a very close genetic relationship. *Plasmodium vivax* came from Africa and, based on genomic data, there is a hypothesis that *P. simium* originated from humans infected with *P. vivax* that was transferred to New World monkeys [8, 12]. There is also an unresolved question of the host transfer and whether *P. brasilianum* in platyrrhines is a result of the cross-species transfer of *P. malariae* that was brought to the New World [3, 13] or whether they are in fact one species [14].

In 2019, Brazil was responsible for 19% of all reported malaria cases in the Americas, and 99.9% of all reported Brazilian malaria cases are from the endemic Amazon region. Furthermore, more than 80% of the cases in Brazil were caused by *P. vivax *[1]. Despite this, there is a substantial number of autochthonous cases reported in the extra-Amazonian region; 1,047 cases were reported in this area between 2006 and 2016 [15]. It is important to highlight that *P. simium* has only been reported in NHPs of the Atlantic Forest on the southern and southeastern states of the Brazilian coast [6, 16], though it can also infect humans, which evidences the exchange of parasitic species between NHPs and humans [9, 11]. Thus, the aim of this study is to screen NHPs in Manaus, in the Brazilian Amazon, for identification of *Plasmodium* infection, including the differential diagnosis of *P. simium*.

## Methods

In a study in Manaus, Amazonas state, Brazil, free-living monkeys were captured in forest fragments inside the urban area using Tomahawk Live (Tomahawk, Wisconsin, USA) traps baited with fruit. Blood samples were collected via femoral vein puncture (1–1.5 mL), and samples were then transported to the laboratory. The animals were maintained in cages and were held overnight and then released in the early morning of the next day at the site of capture. It is important highlight that all animals seemed healthy and none appeared to be in need of veterinary medical assistance. Captive monkeys from a local zoo also had blood samples taken.

It was possible to carry out blood smears for 40% of the samples, but all samples had DNA extraction from blood using the QIAamp DNA Blood Mini Kit (Qiagen, Hilden, Germany), according to the manufacturer’s instructions. The molecular diagnosis was performed using nested PCR targeting the 18 S small subunit (SSU) rRNA and the mitochondrial *coxI* gene [15, 17]. The nested PCR reactions targeting the 18 S SSU used the protocol and primers described by Snounou et al. for diagnosis of *Plasmodium* species infecting humans [17]. The primers described for *P. malariae* were employed to identify *P. brasilianum* infections in NHPs and the primers for *P. vivax* were used to identify *P. simium* infections in NHPs, since these primers do not discriminate among these two pairs of *Plasmodium* species. The differential diagnosis of *P. simium* in relation to *P. vivax* was based on the nested PCR of the *coxI* gene fragment and subsequent enzymatic digestion, using primers and the protocol described by Alvarenga et al. [15].

In the PCR assays, the following were used as positive controls: (i) *P. falciparum* DNA from 3D7 strain in vitro culture (IRR-FIOCRUZ MINAS) [15]; (ii) *P. vivax* DNA, from a patient previously diagnosed by microscopy and nested PCR [15]; (iii) *P. simium* DNA from an acute infection in an NHP with parasitaemia confirmed by optical microscopy (BL10) [18]; (iv) *P. brasilianum* DNA from the Malaria Research and Reference Reagent Resource Center (MR4–ATCC, USA). The negative control was from non-human primates from areas without malaria transmission.

DNA sequencing was performed as described by Alvarenga et al. [15], using mitochondrial *coxI* gene PCR products, which resulted in sequences that covered both single nucleotide polymorphisms (SNPs) that are considered specific to *P. simium* [15]. *Plasmodium simium* differs from the closely related *P. vivax* in two unique single nucleotide polymorphisms (SNPs) in the mitochondrial genome, at positions 3535 (T > C) and 3869 (A > G) [18]. The differential diagnosis of *P. simium* in relation to *P. vivax* was based on the nested PCR of a *coxI* gene fragment and subsequent enzymatic digestion, using the primers and the protocol described by Alvarenga et al. [15].

The polymorphism at position 3535 generated a new restriction enzyme site for HpyCH4III, and the digestion of the amplified fragment resulted in two fragments of similar lengths (118 and 126 bp), where “T” in *P. vivax*, as well in all other *Plasmodium* species tested, is substituted by a “C” in *P. simium* [15].

The fragments were electrophoretically separated in an automatic DNA sequencer (ABI 3730, ThermoFisher). Sequences were aligned using the ClustalW software in the Bioedit package [19] and Chromas software [20].

Evolutionary history was inferred by using the maximum-likelihood method and the Tamura-Nei model [21]. The proportion of sites in which at least 1 unambiguous base is present in at least 1 sequence for each descendent clade is shown next to each internal node in the tree. Evolutionary analyses were conducted in MEGA X [22].

## Results

**Fig. 1:**
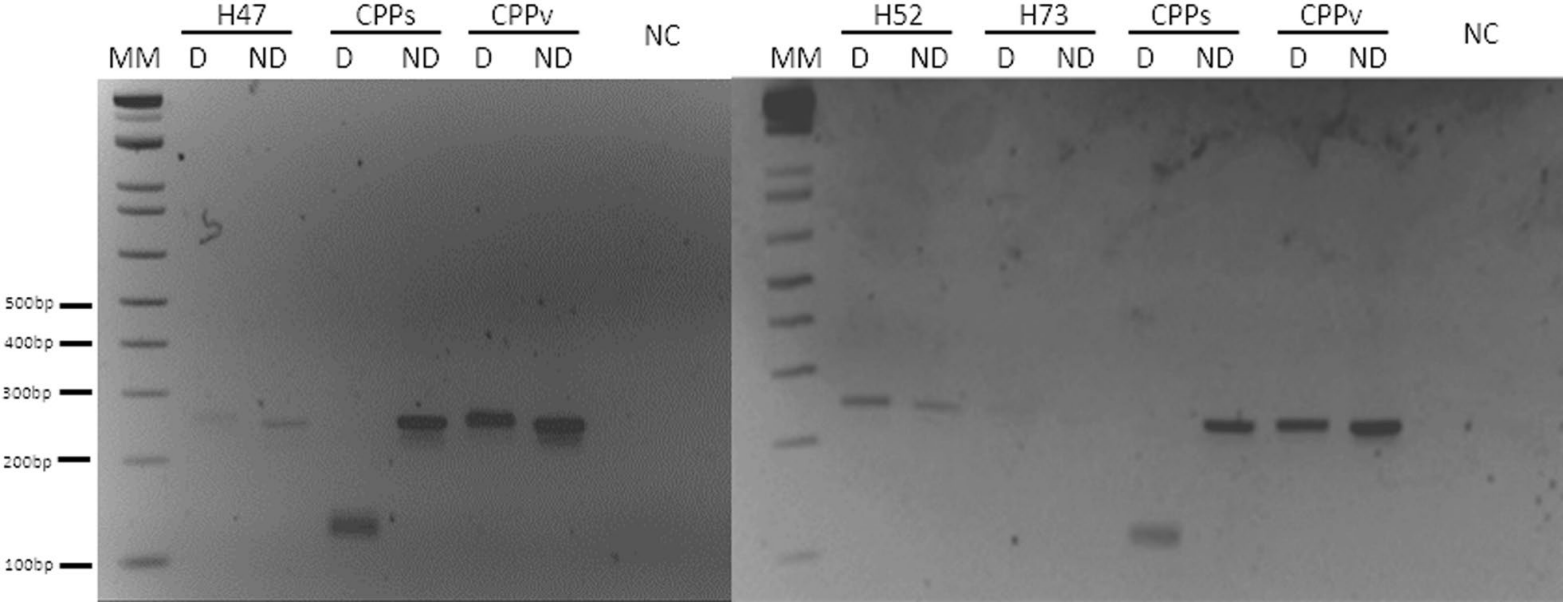
Differential diagnosis of *Plasmodium simium* infection using nested PCR followed by a digestion with HpyCH4III restriction enzyme. H47 captive *Saimiri sciureus*, H52 and H73 free-living *Saguinus bicolor*. 3% agarose gel stained with ethidium bromide. MM:1 kb Plus Ladder (ThermoFischer). D: Digested PCR Product; ND: Undigested PCR product; PCPs: *Plasmodium simium*; PCPv: *Plasmodium vivax*; NC: Negative Control

**Fig. 2:**
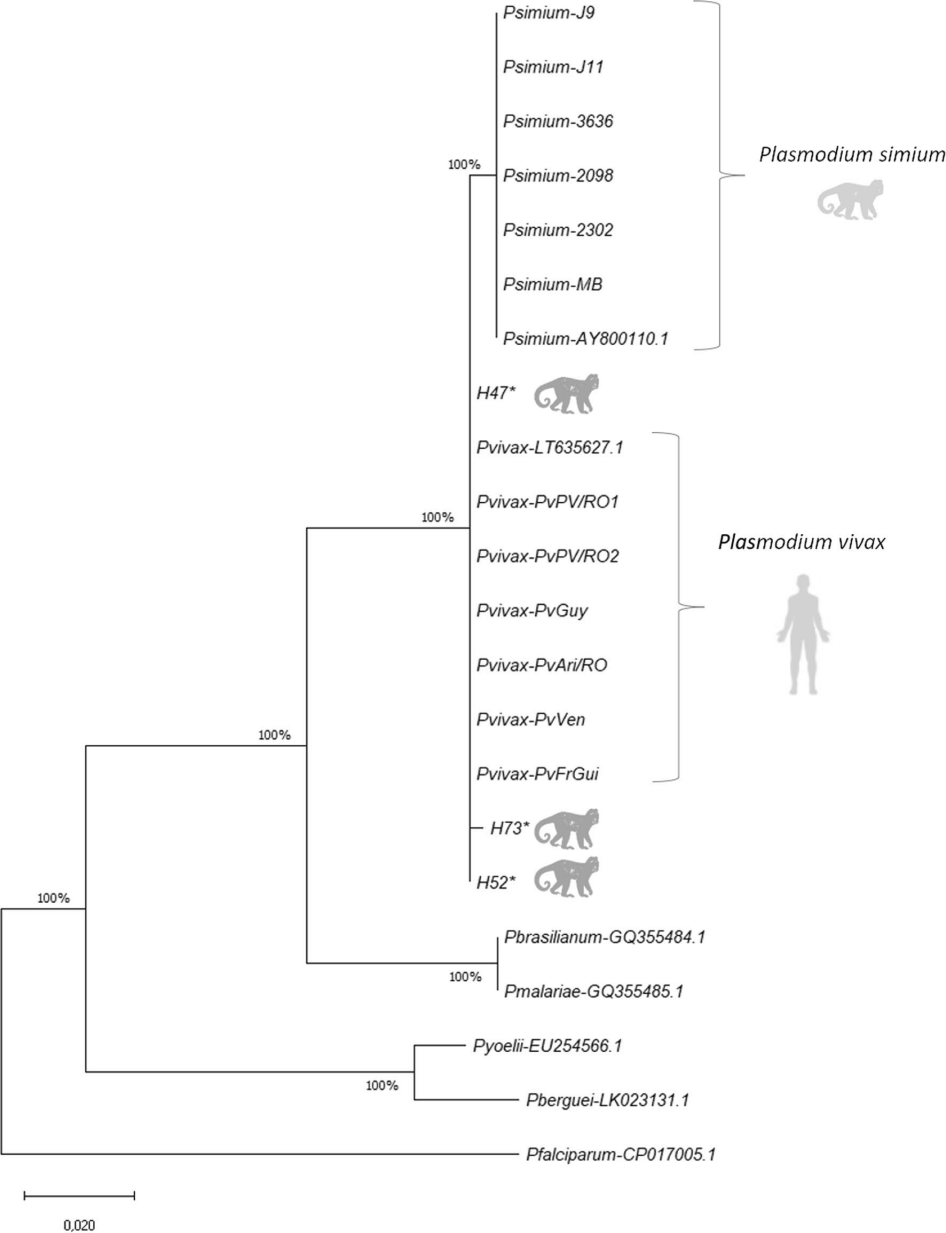
Phylogenetic tree constructed using the maximum-likelihood method with partial mitochondrial sequences of *Plasmodium* isolates. *Plasmodium vivax* isolated from NHPs from Brazilian Amazonia: two of them *Saguinus bicolor* (H52 and H73) and one *Saimiri sciureus* (H47); *P. vivax* isolated from human from Amazon region: PvPV/RO1 and PvPV/RO2 (Porto Velho, Rondonia), PvGuy (Guyana), PvAri/RO (Ariquimedes, Rondônia), PvVen (Venezuela), PvFrGui (French Guiana); *P. simium* isolated from captive (2098, 2302, 3636) and free living NHPs (J9, J11, MB) from Atlantic forest. All *P. simium* and *P. vivax* sequences used here were sequenced by Alvarenga et al. 2018. Accession number at Genbank sequences from *P. simium, P. vivax, P. brasilianum, P. malariae, P. falciparum, Plasmodium berghei* and *Plasmodium yoelii* are included in the name of each sequence. The three new sequences obtained here are marked by an asterisk. Figures represent whether the host of each isolate is a human or a non-human primate

None of the blood smears were positive in the microscopy examination. DNA from 88 samples obtained from 68 NHPs (20 samples were from recapture) (Table 1) was screened for *Plasmodium* spp. using two nested PCR methods. There was no amplification for the 18 S gene; however, three samples (4.4%) were positive in the nested PCR for the *coxI* mitochondrial gene of *Plasmodium* spp, of them only one had blood smears carry out and analysed but was negative. After restriction enzyme digestion, the profile of the fragments suggested non-*P. simium* samples (Fig. 1). The *P. vivax* infection was confirmed using nested PCR/RFLP and sequencing, alignment of partial mitochondrial cytochrome c oxidase subunit I (*coxI*) gene sequences of *P. vivax* isolated from one captive (H47) and two free-living NHPs had 100% identification with GeneBank sequences from *P. vivax* (MG571499.1; MG571498.1) (Fig. 2). Three out of the 68 NHPs, which were two free living *Saguinus bicolor* from the forest fragment of the Federal University of Amazonas (UFAM)—one male (H52) and one female (H73), and one captive *Saimiri sciureus* female (H47) from the army zoo—Centro de Instrução de Guerra na Selva (CIGS) (Fig. 2). The first two had two samples collected at different points in time, but just one sample of each was positive. (H52) was captured for the first time in October 2018 and recaptured in July 2019, and only the first sample was positive. (H73) was captured for the first time in June 2019 and the second sample, which was collected 46 days later, was positive.

**Table 1:** Non-human primates included in the study

Species	Free-living or captive	Location	Year of samples collection	No. of individuals	No. Male	No. Female	*No. positives in Plasmodium* diagnosis
*Saguinus bicolor*	Free-living	UFAM	2018/2019	51*	26	25	2
*Saguinus bicolor*	Free-living	INPA	2018	3	1	2	0
*Saguinus bicolor*	Free-living	Mindu	2018/2019	9	6	3	0
*Saguinus bicolor*	Free-living	CIGS	2018	3	2	1	0
*Pithecia pithecia*	Free-living	INPA	2018	1	1	0	0
*Pithecia pithecia*	Free-living	UFAM	2019	1	0	1	0
*Saimiri sciureus*	Free-living	UFAM	2018	1	1	0	0
*Saimiri sciureus*	Captive	CIGS	2018	3	1	2	1
*Ateles belzebuth*	Captive	CIGS	2018	5	2	3	0
*Ateles chamek*	Captive	CIGS	2018	2	1	1	0
*Ateles paniscus*	Captive	CIGS	2018	2	0	2	0
*Cebus kaapori*	Captive	CIGS	2018	1	1	0	0
*Lagothrix lagotricha*	Captive	CIGS	2018	3	2	1	0
*Sapajus apella*	Captive	CIGS	2018	2**	0	2	0
*Sapajus libidinosus*	Captive	CIGS	2018	2	2	0	0
Total				68	46	43	3

*Twenty were recaptured specimens; **Re-sampled

## Discussion

Three samples amplified from Neotropical NHPs using nested PCR of *coxI* gene fragments were sequenced in both strands, and the consensus sequences were compatible with *P. vivax* species. This is an important finding when trying to evaluate zoonotic vivax infection, and may help us to begin to understand the real role of NHPs in malaria transmission in the Americas. In 2019, in the western Brazilian Amazon, Silva et al. detected *P. vivax* and *P. falciparum* DNA in 2.04% (2/98) and 4.08% (4/98), respectively, of Neotropical primates in captivity, using another protocol [23]. These authors described two individual *Saguinus bicolor* were tested, and one of them was positive for *P. falciparum*, however 12 *Saimiri sciureus* were screened for *Plasmodium*, but none were positive [23]. In a study carried out in 1966, using intracardiac or subcutaneous inoculation, three *Saimiri sciureus* were experimentally infected with the pooled sporozoites from three specimens of *Anopheles cruzi*, though all of them were negative on daily examination of thick blood smears [11].

In Colombia, molecular analysis found Neotropical NHPs that were positive for *Plasmodium* spp.; *P. falciparum* was detected in two fecal samples of *Alouatta seniculus*, while *Cebus versicolor*, *Ateles hybridus* and *Alouatta seniculus* were infected with *P. vivax/simium*, and these last two species and *Aotus griseimembra* had fecal samples that were positive for *P. malariae/brasilianum*. Blood samples were also tested in this study, and one *Ateles hybridus* and one *Alouatta seniculus* were positive for *P. vivax/simium*, while these same two species plus *Aotus griseimembra* and *Cebus versicolor* were positive for *P. malariae/brasilianum* [24]. The present paper shows novel results in regards to the species *Saguinus bicolor.* These are important since, in the majority of previous studies, positive results for *P. vivax* in NHPs were only serological finds and indistinguishable positive PCR results for *P. vivax/P. simium* [25, 26].

*Plasmodium vivax* and *P. simium* can be misdiagnosed due to their morphological and molecular similarity. Malaria caused by *P. simium* is apparently restricted to regions of the Atlantic Forest on the coast of southeastern and southern Brazil [6]; despite this, it was possible to identify three NHPs with *P. vivax* using differential diagnosis. Identification of human malaria parasites in NHPs in the north of this country gets deserves attention since a zoonotic cycle for *P. vivax* in the Americas has not yet been considered due to a lack of scientific evidence. Sampling was performed in Manaus, capital of the Amazonas state and located in the middle of the tropical rainforest (− 3.044653 S, − 60.1071907 W), where more than 2 million people coexist with malaria. Besides socioeconomic difficulties, there are nuances in vivax malaria that complicate all efforts towards malaria elimination in this region, such as its hypnozoite form that causes late relapses, the existence of drug resistant parasites [27] and the loss of social importance since people have become used to living with the disease [28]. It may be that the animal reservoir could be one more obstacle for malaria elimination.

The richness of Neotropical primate species (Platyrrhini) is evidenced by the 171 species in 20 genera and five families [29]; however, there is a big knowledge gap in relation to which species can be infected with *Plasmodium* species, for how long and their importance in the parasite cycle. In addition, the presence of parasites in the blood of NHPs demands attention and discussion, since NHPs represent an animal reservoir in the vivax malaria cycle and, as such, the risk this presents should return to the discussion agenda.

Three primates (two free living and one captive) that were captured in small forest fragments in the urban area were *P. vivax* positive. In another study, the presence of two *Anopheles* species was identified at the CIGS zoo, namely *Anopheles matogrossensis* and *Anopheles nimbus*, although they are not species associated with human malaria transmission, which reinforces the results present here and strengthens the hypothesis of a zoonotic cycle. In addition, *An. nimbus* and *Anopheles triannulatus* were identified at UFAM [30]. The latter, *An. (Nysorhynchus) triannulatus*, is considered to be a secondary human malaria vector in some areas in Brazil [31, 32] and a dominant vector in the east of Loreto, Peru [33]. These areas in which vectors and potential vectors were collected (CIGS and UFAM) are the same areas that the primates were found to be positive for *P. vivax* between 2018 and 2019.

Since *Saguinus bicolor* is a critically endangered species [34], it is necessary to carry out studies aimed at the impacts that *Plasmodium* infection can cause on animal health and the conservation of this primate species, which is endemic to Manaus [35].

## Conclusion

The data shown here reinforce and bring into question again the possibility of a non-human reservoir of *P. vivax* in the Amazon, which is worrisome since *P. vivax* is the agent of the greatest number of cases of malaria in the Americas. Knowledge regarding the disease cycle is essential in order to plan measures for mitigation, as well as for defining targets that help us to achieve the ultimate goal, which is, of course, malaria elimination. The assessment of the frequency of *P. vivax* infection in NHPs and the evaluation of the ecological importance of these primates as the parasite’s reservoir are urgent measures, and the questions presented herein need to be included in the agenda of further studies. As such, it remains to be determined whether wildlife management as a component of malaria elimination programmes is necessary.

## Data Availability

Data analysed during this study is included in this published article and dataset used and/or analysed during current study are available from corresponding author on demand.
